# Factors Affecting Thai Fathers’ Self-Efficacy to Support Exclusive Breastfeeding

**DOI:** 10.3390/nursrep13040127

**Published:** 2023-10-27

**Authors:** Preeyakamon Krikitrat, Nantaporn Sansiriphun, Jirawan Deeluea, Sirirat Sonted, Wongduean Chaiwipassatorn, Daniel Bressington

**Affiliations:** 1 Department of Obstetrics and Gynecology Nursing, Faculty of Nursing, Chiang Mai University, 239 Huay Kaew Rd, Tambon Su Thep, Amphoe Mueang Chiang Mai, Chang Wat Chiang Mai 50200, Thailand; preeyakamon.lert@cmu.ac.th (P.K.); nantaporn.san@cmu.ac.th (N.S.); jirawan.d@cmu.ac.th (J.D.); sirirat.sonted@cmu.ac.th (S.S.); 2 Maharaj Nakorn Chiang Mai Hospital, Faculty of Medicine, Chiang Mai University, Amphoe Mueang Chiang Mai, Chang Wat Chiang Mai 50200, Thailand; wongduen.chaiwip@cmu.ac.th; 3 Visiting Professor, Faculty of Nursing, Chiang Mai University, Amphoe Mueang Chiang Mai, Chang Wat Chiang Mai 50200, Thailand; 4 Faculty of Health, Charles Darwin University, Ellengowan Drive, Casuarina, NT 0810, Australia

**Keywords:** fathers, self-efficacy, exclusive breastfeeding, affecting factors

## Abstract

Background: Breastfeeding is the ideal approach for feeding infants and is an important public health consideration. Successful exclusive breastfeeding initiation and duration is influenced by fathers’ support. Paternal self-efficacy to support breastfeeding has also been shown to mediate infant feeding practices. The aim of this study was to investigate factors associated with Thai fathers’ self-efficacy to support maternal exclusive breastfeeding. Methods: We adopted a cross-sectional survey design for this study. In total, 215 Thai fathers who had a partner with a term (37–42 weeks) pregnancy participated in the study. Data were collected from antenatal care clinics at two hospitals in Northern Thailand between June and August 2022. Participants completed a sociodemographic questionnaire, the Fathers’ Attitude toward Exclusive Breastfeeding questionnaire, the Fathers’ Knowledge about Exclusive Breastfeeding questionnaire, and the Breastfeeding Self-Efficacy Scale (Short-Form). Multiple linear regression and hierarchical regression were used to analyze factors influencing Thai fathers’ self-efficacy to support maternal exclusive breastfeeding. Results: The mean breastfeeding self-efficacy score was 52.94 (*SD* = 8.58), indicating that fathers were confident they were able to support their partners’ breastfeeding. Regression analysis revealed family type, fathers’ attitude toward, and fathers’ knowledge about exclusive breastfeeding significantly explaining 14.90% of the variance in paternal breastfeeding support self-efficacy. However, fathers’ age, education, employment, income, and number of living children were not associated with their self-efficacy. Conclusions: The results demonstrated that family type and fathers’ attitudes/knowledge about breastfeeding influenced their self-efficacy to support exclusive breastfeeding. Nurses should consider implementing breastfeeding interventions specific to fathers to enhance their attitudes and knowledge about breastfeeding, including increasing fathers’ self-efficacy to support maternal exclusive breastfeeding efforts.

## 1. Introduction

Breastmilk includes all the nutrients required to promote the health of infants and enhance their immune systems [1]. The World Health Organization (WHO) recommends that infants should be fed only breastmilk (without water or food) until the child is at least 6 months old, and breastfeeding should continue (with food and water) until at least 2 years of age [2]. Infants who breastfeed have lower risks of respiratory infections, diarrhea, and injury from nutritional deficiencies that impair cognitive development [3,4]. In addition, breastfeeding benefits mothers by reducing their risk of postpartum hemorrhage and protecting against breast and ovarian cancers [5,6,7].

Thailand accepted the World Health Organization’s breastfeeding recommendations, and, subsequently, exclusive breastfeeding has been heavily promoted nationwide. However, the country has made inadequate progress towards improving breastfeeding practices. In 2007, the exclusive breastfeeding rate in Thailand (5.4%) was the third lowest globally and the lowest in Southeast Asia [8]. Although Thailand experienced an increase in the exclusive breastfeeding rates from 12.3% in 2013 [9] to 23.1% in 2016, these rates are still lower than those reported by other Southeast Asian countries [10]. Unfortunately, the most recent evidence showed that Thailand’s exclusive breastfeeding rate rapidly decreased to 14% in 2019 [11], which is well below country’s exclusive breastfeeding goal of 50% for the year of 2021 (National Statistical Office and United Nations Children’s Fund, 2016) [10].

To date, Thai researchers have primarily focused their breastfeeding studies on the perspectives and behaviors of mothers, but this work seems to have only had a limited impact on meeting breastfeeding targets. Successful breastfeeding is related to a convergence of factors, including individual factors, sociocultural issues, and public health polices [12]. It is important not to overlook the fact that fathers can influence exclusive breastfeeding rates because they provide social and practical support when their partners are breastfeeding their child [13]. Previous studies have shown that higher rates of maternal self-efficacy for breastfeeding are related to greater levels of paternal support and encouragement [14]. Mothers whose partners were supportive have also reported feeling more able and confident to overcome breastfeeding setbacks and make breastfeeding decisions [15]. Indeed, researchers who have conducted studies in Canada, South Africa, and Australia have shown that higher levels of fathers’ support are related to better breastfeeding practices, including both breastfeeding initiation and duration [14,16,17]. Fathers engage in a range of social and childcare support activities following the birth of their children, including parenting and taking on household work to share the load [18]. Moreover, fathers also help to motivate and assist their partners to attend healthcare appointments in addition to providing financial and emotional support [17].

Although results of many studies have shown that fathers have an interest in breastfeeding and are keen to be involved, studies have also found that they did not feel adequately prepared to support their partners breastfeeding [19,20]. Fathers have reported feeling embarrassed, anxious, helpless, and lacking confidence to provide support when their partners breastfeed [19,21] and frequently felt excluded from childcare [21]. This is concerning because mothers are more likely to discontinue breastfeeding when their partners have low levels of confidence in providing support to overcome breastfeeding difficulties [20,21]. Lack of breastfeeding preparation and support for fathers may also lower their quality of life and sense of self-efficacy, which is defined as the perception of their abilities [22].

Dennis et al. demonstrated that self-efficacy for breastfeeding support among fathers related to successful breastfeeding outcomes [23]. Therefore, a better understanding of factors that promote or hinder paternal self-efficacy merits specific exploration in order to better understand its influence on infant feeding practices. Measuring fathers’ self-efficacy would also be useful to predict the extent of their breastfeeding support and guide the development of breastfeeding interventions to increase fathers’ confidence to support their partners’ breastfeeding [24]. Dennis et al. (2018) conceptualized paternal breastfeeding support self-efficacy as a “father’s perceived ability to assist his partner in breastfeeding their infant” and created a standardized measure entitled the Breastfeeding Self-Efficacy Scale-Short Form (BSES-SF) [23]. The psychometric properties of the BSES-SF were reported as sound, and the authors found that Canadian fathers’ paternal BSES-SF scores were significantly associated with their perceived importance of breastfeeding and breastfeeding attitude. Paternal BSES-SF scores were also significantly related to mothers’ perceptions of breastfeeding progress, breastfeeding level and exclusivity [24]. 

In the Thai family context, fathers are traditionally viewed as providers or breadwinners and are removed from the nurturing role fulfilled by mothers. Culturally, consanguineal ties among women in Thailand are significant [25], and women’s relationships with their mothers are tightly connected to their own motherhood [26]. Thus, grandmothers play an important role in the household for maternal breastfeeding practices. Moreover, hospitals do not routinely emphasize fathers’ involvement in breastfeeding [26]. As a result, Thai fathers tend to lack knowledge of breastfeeding support and to show low confidence in their ability to provide support when their partners breastfeed. However, as economic forces are now making nuclear families the norm in Thailand (as opposed to traditional extended families), fathers are increasingly expected to not only play the role of provider but also to be more closely involved in the childrearing process [27]. This shift in role expectations may present challenges for Thai fathers and erode their self-efficacy. Given the unique Thai cultural context studies exploring paternal self-efficacy to support exclusive breastfeeding that were conducted in other countries are not directly generalizable. It is therefore important to conduct culturally and contextually specific studies to investigate the influences on Thai fathers’ self-efficacy to support mothers’ breastfeeding practices. 

To date, only one study has been conducted to examine the relationships between Thai fathers’ self-efficacy to support breastfeeding and exclusive breastfeeding duration, and this was conducted during the postpartum period, which begins after childbirth until six weeks [28]. The study’s results revealed that paternal age, educational level, and fathers’ attitudes toward exclusive breastfeeding were associated with their self-efficacy to support exclusive breastfeeding [28]. However, correlations between paternal sociodemographic characteristics, fathers’ attitude toward exclusive breastfeeding, fathers’ knowledge about breastfeeding, and their self-efficacy to support breastfeeding have not been observed in the antenatal period (i.e., the period from their partners’ pregnancy until the onset of labor). This is an important gap in knowledge because fathers’ levels of self-efficacy will differ between the antenatal and postnatal periods due to the anticipation of childbirth as opposed to the reality of caring for a newborn child. The factors associated with fathers’ self-efficacy to support breastfeeding may also be different between the antenatal and postnatal periods. A better understanding of these issues would help to inform the development of antenatal interventions for fathers. Thus, we used the breastfeeding self-efficacy theory [23] as a conceptual framework. We also examined how the four main sources of self-efficacy identified in Dennis et al.’s (2018) theory—performance accomplishments, vicarious experiences, verbal persuasion, and physiologic responses—explain fathers’ self-efficacy to support their partners’ breastfeeding.

## 2. Materials and Methods

### 2.1. Study Aims and Design 

The aim of the current study was to identify levels of paternal self-efficacy to support maternal exclusive breastfeeding and investigate associated factors. A descriptive cross-sectional survey design was used. The specific research questions and corresponding hypotheses were as follows:

What are the levels of Thai father’s self-efficacy to support exclusive maternal breastfeeding, and which paternal sociodemographic factors are significantly associated with self-efficacy?

**Hypothesis** **1:**  *Fathers’ self-efficacy will be significantly associated with age, education, type of family, employment, income, and number of living children.*

How much is fathers’ self-efficacy to support exclusive maternal breastfeeding explained by sociodemographic factors, fathers’ attitudes toward exclusive breastfeeding, and fathers’ knowledge about exclusive breastfeeding?

**Hypothesis** **2:**  *A statistically significant proportion of fathers’ self-efficacy will be explained by a combination of paternal sociodemographic factors, fathers’ attitudes toward exclusive breastfeeding, and fathers’ knowledge about exclusive breast-feeding.*

### 2.2. Study Setting

Data collection was conducted from the antenatal care clinics of two hospitals in Thailand’s Chiang Mai province from June to August 2022. 

### 2.3. Participants and Data Collection

The study participants consisted of 215 fathers whose partners were at term (gestational age 37–42 weeks) during pregnancy to be eligible for the study, and fathers needed to understand the Thai language and be aged 20 years or older. Fathers whose partners had antenatal complications were excluded because their experiences of pregnancy and early childcare would not be typical for most fathers. These complications include sexually transmitted diseases, hypertension, vaginal bleeding, and mental health problems. In addition, fathers whose partners had no intention to breastfeed were excluded. 

After obtaining approval from institutional review boards, fathers were recruited using a convenience sampling approach, where they were given information flyers in each of the antenatal clinics. The flyers provided the principal investigator (PI)’s contact information and invited fathers who were interested in participating to telephone or email the PI. In addition, at the antenatal care clinics, the PI individually approached fathers and asked them to consider participating in the study. When fathers expressed interest in participation in the study, the PI first confirmed their eligibility, shared written information on the study, and obtained their written informed consent to participate. Once informed consent was received, the PI administered the survey, participants filled out the survey in person at an antenatal care clinic in a private space, and it took between 15 to 30 min complete. After returning the completed survey to the PI, each participant was compensated 100 Thai Baht (3 USD) in cash for their participation. 

### 2.4. Sample Size

A power analysis and information from earlier studies on breastfeeding self-efficacy were used to calculate the sample size [23,28]. In the power analysis using G*power program version 3.1.9.7 with multiple linear regression (mixed model, R^2^ deviation from zero statistics), to estimate the effect size of 0.10 with 8 predictors (age, education, type of family, employment, income, number of living children, fathers’ attitude toward, and fathers’ knowledge about exclusive breastfeeding), a sample size of 215 was needed to obtain the power of 0.80 with an alpha of 0.01.

### 2.5. Quantitative Variables and Measures

Variables from literature reviews and previous studies that showed correlations between demographic variables, attitudes toward exclusive breastfeeding, knowledge about exclusive breastfeeding, and breastfeeding self-efficacy [23,28,29,30] were selected. Permission to use the published outcome measures was obtained from the original authors prior to conducting the study. 

**Sociodemographic variables.** Each participant completed a sociodemographic questionnaire specifically designed for this study, which gathered data on age, education, type of family (nuclear vs. extended), employment, income, and number of living children. A nuclear family was defined as a couple living in their house without any relatives, whereas an extended family was defined as a couple living permanently (as perceived by the father, i.e., the relative was not visiting temporarily) in the household with at least one other extended family member (i.e., parent, grandparent, great-grandparent, aunt, uncle, cousin, brother, or sister). These sociodemographic variables were identified as being potentially important predictors of paternal self-efficacy in previous studies [23,28,29,30]. 

**Fathers’ self-efficacy to support exclusive breastfeeding**. Fathers’ self-efficacy was defined as fathers’ perception of their ability to assist their partners in breastfeeding (Dennis et al., 2018). We used the Breastfeeding Self-Efficacy Scale–Short Form (BSES-SF) for fathers, which was developed by Dennis et al. in 2018 [23] and was previously translated into Thai by Krikitrat et al. [28], to assess fathers’ self-efficacy to support exclusive breastfeeding. The 14-item questionnaire uses a 5-point Likert-type scale for each question (with 1 indicating not at all confident to 5 indicating always confident). Total scores range from 14 to 70, with higher scores indicating more optimal levels of breastfeeding self-efficacy. The BSES-SF for fathers is a specific questionnaire that was modified for assessing fathers’ perception of their confidence to support breastfeeding [23]. It also had acceptable indicators of validity and reliability to assess paternal self-efficacy for breastfeeding support [23,28]. Krikitrat et al. found that the translated BSES-SF for fathers had a Cronbach’s alpha of 0.93, indicating good reliability [28]. The internal consistency of the BSES-SF score in the current study was excellent (Cronbach’s alpha coefficient of 0.91). 

**Fathers’ attitudes toward exclusive breastfeeding**. Fathers’ attitudes toward exclusive breastfeeding referred to fathers’ feelings and opinions about maternal exclusive breastfeeding. The participants in this study completed the Thai-language Father’s Attitude toward Exclusive Breastfeeding questionnaire developed by Howkanta et al. [31]. The questionnaire was developed based on Thai cultural context and focused on assessing Thai fathers’ feelings or opinions about breastfeeding. The questionnaire comprises 31 five-point Likert-type scale questions, each scored from 1 (strongly disagree) to 5 (strongly agree). Higher scores indicate a more positive paternal attitude toward exclusive breastfeeding. The questionnaire is reported to have excellent internal reliability (Cronbach’s alpha of 0.93) when used with Thai fathers [31]. In the current study, the Cronbach’s alpha coefficient was 0.86, indicating good internal consistency.

**Fathers’ knowledge about exclusive breastfeeding**. Fathers’ knowledge about exclusive breastfeeding referred to fathers’ familiarity with information about exclusive breastfeeding or their understanding of this information. The participants completed Howkanta et al.’s Thai-language version of the Fathers’ Knowledge about Exclusive Breastfeeding questionnaire [31]. The questionnaire consists of 34 items that are rated true, false, and do not know. This instrument was selected because it covers important facets of exclusive breastfeeding and has items that are relevant to the Thai context. The questionnaire includes 18 items of benefits of breastfeeding, 11 items of breast milk’s nutrients, and 5 items of duration of breastfeeding and supplementary food feeding. Total scores range from 0 to 34, with higher scores indicating more knowledge about exclusive breastfeeding. In Howkanta et al.’s study, the questionnaire had a Cronbach’s alpha of 0.84 [31]. The Cronbach’s alpha coefficient estimate of internal consistency in the current study was 0.83.

### 2.6. Pilot Testing

We conducted an initial pilot study of the survey with ten Thai fathers that were not included in the final 215 participants. The results revealed that there were no required changes to the questionnaires. The Cronbach’s alpha values of the Breastfeeding Self-Efficacy Scale–Short Form, Fathers’ Attitude toward Exclusive Breastfeeding questionnaire, and Fathers’ Knowledge about Exclusive Breastfeeding questionnaire were 0.95, 0.87, and 0.85, respectively. 

### 2.7. Ethical Considerations

Approvals to conduct the study were prospectively obtained from the University’s ethics committee (#2022-EXP020) and the two hospitals (#NONE-2565-09062 and #24/2565). The study flyer stated the purpose of the study, the voluntary nature of participation, inclusion criteria, and benefits and risks of participation. The PI discussed this information with potential participants and ensured that they fully understood and were able to provide informed consent. After answering any of the participants’ questions about the study, the PI obtained their signed informed consent. 

### 2.8. Statistical Analysis

Descriptive statistics was used to describe participant’s sociodemographic information (age, education, type of family, employment, income, and number of living children), as well as scores for the BSES-SF for fathers, the fathers’ attitude toward exclusive breastfeeding, and the fathers’ knowledge about breastfeeding. Frequencies/percentages were calculated for categorical variables, whereas means and standard deviations were computed for continuous variables. There were no missing data, as the questionnaires were completed by fathers in the presence of a researcher who was able to check that all items had been completed.

The assumptions of data were tested for variables in the regression models. The results showed that no assumptions were violated, including normality of dependent variable, linear relationships, significant outliers, multicollinearity, residual normal distribution, homoscedasticity, or autocorrelation. Therefore, we employed an initial multiple linear regression analysis to estimate the relationships between fathers’ self-efficacy to support exclusive breastfeeding (dependent variable) and paternal sociodemographic factors (independent variables) to test hypothesis 1. For paternal sociodemographic factors, the categorical variables were recorded into a number of separate and dichotomous variables. The reference groups were unemployment, nuclear family, bachelor’s degree and higher education, and number of children > 1. 

A hierarchical multiple regression model was finally used to identify factors influencing father’s self-efficacy to support exclusive breastfeeding, hence testing hypothesis 2. In the analysis, we included significant sociodemographic factors identified in the initial exploratory regression model and added fathers’ attitude toward breastfeeding, and fathers’ knowledge about breastfeeding in two stages. The data were analyzed using statistical software (Stata License version 15 no.: 301506232609)(StataCorp LLC., College Station, TX, USA). A *p*-value of <0.05 was considered statistically significant for all analysis (two-sided).

## 3. Results

### 3.1. Socio-Demographic Characteristics

In total, 223 fathers met the study’s inclusion criteria. Eight fathers declined to participate due to limited availability. Thus, 215 fathers completed the survey. The participants’ socio-demographic characteristics are shown in Table 1.

Participants ranged from 20 to 52 years of age (*M* = 31.62, *SD* = 6.26). Most participants had completed less than a bachelor’s degree. More than half lived in extended family households and earned 10,000–19,999 Baht (300–600 USD) per month (*M* = 17,498.36, *SD* = 9469.39). Most participants were employed. About three-quarters of participants were first-time fathers.

### 3.2. Father’ Self-Efficacy, Attitude towards Exclusive Breastfeeding, and Knowledge about Breastfeeding

Participants’ mean self-efficacy score was 52.94 (*SD* = 8.58), with scores that ranged from 20 to 70. Participants’ mean attitude towards exclusive breastfeeding score was 120.74 (*SD* = 11.71), with scores that ranged from 83 to 148. Participants’ mean knowledge about breastfeeding score was 17.38 (*SD* = 5.74), with scores that ranged from 2 to 31. 

### 3.3. Sociodemographic Factors Associated Thai Fathers’ Self-Efficacy to Support Exclusive Breastfeeding

The results of the initial multiple linear regression indicated that only family type was significantly associated with paternal self-efficacy to support exclusive breastfeeding (*p* < 0.05). However, there was no relationship between fathers’ age, education, employment, income, and number of living children and their self-efficacy (Table 2).

### 3.4. Factors Affecting Thai Fathers’ Self-Efficacy to Support Exclusive Breastfeeding

Hierarchical multiple regression was conducted using three levels. In the first model, type of family was a significant predictor and accounted for the 3.4% of total variance (R^2^ = 0.034, Adjusted R^2^ = 0.03, F = 7.49, *p* < 0.05). Extended family was significantly negatively associated with paternal self-efficacy to support exclusive breastfeeding (β = −3.38, *p* < 0.05). In the second model, the results showed a statistically significant relationship between the explanatory variables and the level of fathers’ self-efficacy to support exclusive breastfeeding, predicting 13.2% of score variability (F = 16.09, *p* < 0.05). The explanatory variance increased by 9% from model 1 to model 2. Fathers’ attitude toward exclusive breastfeeding was found to be a significant predictor of self-efficacy (β = 0.24, *p* < 0.05), indicating that more positive paternal attitude was significantly and positively associated with their self-efficacy. In model 3, which included fathers’ knowledge about exclusive breastfeeding, three factors explained 14.90% of the variance in paternal self-efficacy to support exclusive breastfeeding (F = 12.30, *p* < 0.05), which indicated that fathers’ knowledge increased the explanatory variance by 2%. Fathers’ knowledge about breastfeeding remained significantly associated with fathers’ self-efficacy to support breastfeeding in model 3 (β = 0.22, *p* < 0.05) (Table 3). 

## 4. Discussion

The study results demonstrated that participants had confidence in their ability to support their partners’ breastfeeding. Similarly, another study that surveyed 205 Thai fathers with 6-month postpartum partners found a mean score of 54.65 on breastfeeding support self-efficacy [28]. Although this study was conducted in the antenatal period, whereas the other one was conducted in the postpartum period, the similarity in the mean BSES scores between the two studies could be due to several reasons. First, the majority of participants in both studies were relatively affluent (earned 10,000–19,999 Baht (300–600 USD)/month), worked full-time, had a similar average age, and were first time fathers. These similar characteristics may give them confidence to support their partners’ breastfeeding. Second, the two studies were conducted in the same location, Chiang Mai, Thailand, where fathers may have similar experiences of clinical services and shared perceptions of the culture and social climate supporting breastfeeding. Third, the participants in our study were fathers whose partners were between 37 and 42 weeks of gestation, which indicated that they were close to delivery, and the outcomes may not have varied significantly in the postpartum period.

The study’s findings also revealed a significant relationship between the type of expectant fathers’ family and paternal self-efficacy to support exclusive breastfeeding. Previous studies have not reported similar relationships between paternal sociodemographic characteristics during the antenatal period and paternal self-efficacy. Interestingly, although more than half the fathers in the current study lived in extended families, living with other family members was found to be negatively associated with paternal self-efficacy to support their partners’ breastfeeding. This result contrasted with the Thai traditional family context that family members, especially grandmothers, were viewed as key persons influencing on mother’s breastfeeding [32,33,34]. Most Thai grandmothers are well experienced in breastfeeding, so family members may have relied on grandmothers for this task and followed grandmother’ suggestions about breastfeeding, which may reduce levels of paternal self-efficacy to support exclusive breastfeeding [35].

Indeed, previous studies conducted in Papua New Guinea, Guatemala, and Mexico have reported that grandmothers negatively influenced breastfeeding duration and exclusivity, which may provide some explanation for the current study’s findings [36,37,38]. Grandmothers’ attitudes towards and understanding of breastfeeding may present problems beyond nutritional health due to a lack of knowledge about current evidence-based breastfeeding practices, such as the early introduction of complementary foods to infants less than six months old [36,38]. For example, the study conducted in Papua New Guinea reported that grandmothers administered water or tea to the grandchildren, which caused them to cease exclusively breastfeeding [36]. Moreover, grandmothers exerted a dominant influence on the breastfeeding practices of mothers in both Papua New Guinea and Brazil [36,39]. Therefore, the lower breastfeeding support confidence of fathers in extended families may be caused by social influence and fathers’ perceptions of grandmothers’ (or other influential extended family members’) control over infant feeding within their household. 

Despite the hierarchical multivariable regression model being statistically significant, the independent variables only explained 14.9% of the variance in paternal self-efficacy scores, highlighting that other unmeasured extraneous variables may have important influences on fathers’ self-efficacy to support breastfeeding. In attempting to identify other predictors of fathers’ self-efficacy, relationships between Thai fathers’ sociodemographic variables and their self-efficacy were examined in only one previous study. Krikitrat and colleagues found that paternal self-efficacy was significantly and positively related to age and educational level, possibly suggesting that older well-educated fathers may have a better grasp of breastfeeding information, and this enhances their self-efficacy to provide breastfeeding support [28]. However, we found no such associations between these two variables in the current study. A potential explanation is that Thai fathers currently learn about breastfeeding from various sources such as family members, friends, colleagues, neighbors, healthcare providers, community members, and social media [33,40,41]. Therefore, fathers within all age and education levels could expand their understanding of breastfeeding to increase their self-efficacy from outside sources. Maternal self-efficacy is another important potential influence on fathers’ self-efficacy to support breastfeeding that was not measured in the current study. Previous studies have shown that paternal self-efficacy was significantly correlated with their partners’ self-efficacy during the postpartum period [23,28]. Other potential influences on paternal self-efficacy that were not measured in this study include the type and quality of support from healthcare professionals, previous personal experience with breastfeeding, and cultural norms [42,43].

The results of our study also suggest that fathers’ positive attitudes toward exclusive breastfeeding are positively associated with their breastfeeding support self-efficacy. A similar finding was reported by Dennis et al. in Canada, where paternal BSES-SF scores immediately following birth and at 6 weeks postpartum were found to be significantly correlated with their exclusive breastfeeding attitude [23]. Moreover, in a previous study of 205 Thai fathers, Krikitrat and colleagues reported that paternal self-efficacy was significantly correlated with their attitude toward exclusive breastfeeding in the 6 months postpartum [28]. Fathers’ attitude toward breastfeeding was also positively related to their involvement in breastfeeding [44].

Additionally, we found a significant association between fathers’ self-efficacy and their knowledge about breastfeeding in this study. No such correlations were observed in previous studies. Although the magnitude of the relationship between these two variables was small (β = 0.22, *p* < 0.05), and the result should be interpreted with caution, it is possible that improving either fathers’ self-efficacy or knowledge may have a positive impact on the other variable. For example, a father’s relatively low self-efficacy may relate to their perceptions of having inadequate knowledge. However, given the lack of evidence in the area and the preliminary findings of a small effect size in our study, subsequent studies should consider measuring how the relationship between fathers’ knowledge and self-efficacy may change over time. Among mothers, correlations between self-efficacy and breastfeeding knowledge are common [45,46], for example, Corby and colleagues found that breastfeeding knowledge was a significant positive predictor for Canadian mothers’ self-efficacy in the prenatal period [45]. According to Corby’s study, healthcare professionals can strategically target women who are at risk of having low breastfeeding self-efficacy and provide an early intervention to support breastfeeding [45]. 

Finally, regression analysis indicated that being in a nuclear family, fathers’ attitude toward exclusive breastfeeding, and fathers’ knowledge about breastfeeding were associated with paternal breastfeeding supported self-efficacy. These results seemed to highlight that interventions designed to promote paternal breastfeeding support self-efficacy would benefit from promoting the engagement of both fathers and nuclear family members in the exclusive breastfeeding process. It is also possible that improving fathers’ knowledge may result in increased self-efficacy to support their partner’s exclusive breastfeeding. Indeed, some previous intervention studies have reported improvements in paternal breastfeeding support self-efficacy scores. For example, a Canadian randomized controlled trial of a co-parenting intervention showed a significant improvement in paternal breastfeeding support self-efficacy scores from baseline to 6 weeks postpartum (*p* = 0.03) [16]. Moreover, Kiyaeimolasaraei and colleagues’ Iranian study found that fathers who attended antenatal classes scored higher on paternal breastfeeding self-efficacy than those who did not (*p* = 0.003) [46]. The findings also revealed a significant correlation between paternal breastfeeding self-efficacy score and paternal cooperation in prenatal care (*p* = 0.001). Participation in prenatal care and antenatal classes by the fathers can increase paternal self-efficacy to support breastfeeding [47]. Similarly, providing support to influential extended family members (such as grandmothers) may also be a benefit. Previous studies conducted in Canada and Indonesia reported that fathers and grandmothers gained knowledge and positive attitudes towards breastfeeding through antenatal classes and programs that provided guidance on how to support breastfeeding to mothers [19,48]. 

## 5. Implications

Interventions that aim to improve fathers’ self-efficacy and enhance their involvement in breastfeeding support should be inclusive and promote co-parenting. Future interventions should also consider including other influential family members to enable them to provide maternal breastfeeding support and increase paternal self-efficacy. For further longitudinal studies, fathers’ self-efficacy and its factors should be examined both in the antenatal and postpartum periods to determine changes in paternal confidence in their capacity to support exclusive breastfeeding after childbirth, when fathers have experienced supporting their partner following birth. Also, specific subpopulations of fathers, such as fathers whose partner or child had postpartum complications and teenage fathers, should be studied in order to better understand paternal self-efficacy. Moreover, assessing alternative family configurations or family types can help identify structural family patterns that are most influential to paternal self-efficacy to support their partners’ breastfeeding. The findings would expand our understanding of paternal factors that impact fathers’ self-efficacy to support exclusive breastfeeding by providing breastfeeding support under a variety of circumstances, which, in turn, would help to design interventions to raise paternal self-efficacy and possibly improve Thailand’s exclusive breastfeeding rate. Finally, it would be worthwhile exploring the lived experience of Thai fathers in their efforts to support exclusive breastfeeding, as this may help to better understand the significant associations identified between quantitative variables in the current study and identify specific Thai cultural nuances that may not be captured by the survey questionnaires. 

## 6. Limitations

Our study findings have limited generalizability due to the study’s design and sampling methods, and we are unable to demonstrate causative relationships. Moreover, we only collected data in the antepartum period, so the full spectrum of paternal self-efficacy was not captured. In addition, although the Thai language version of the survey instrument was validated, no additional items or cultural modifications were suggested by the participants, potentially resulting in a questionnaire that may not fully capture the nuances of Thai breastfeeding culture. Finally, the study variables only accounted for 14% of the variance in paternal self-efficacy to support maternal exclusive breastfeeding scores, indicating other unidentified influences on father’s self-efficacy that were not captured in the current study. Despite these limitations, this study is the first to evaluate Thai fathers’ self-efficacy to support exclusive breastfeeding in the antenatal period, providing useful information that could be utilized to inform support and education programs. 

## 7. Conclusions

In summary, family type, attitude toward exclusive breastfeeding, and knowledge about breastfeeding were factors affecting Thai fathers’ self-efficacy to support exclusive maternal breastfeeding. The results of this study suggest that paternal breastfeeding support self-efficacy should be promoted in breastfeeding programs. In addition, nurses should implement breastfeeding interventions specific to fathers during the antenatal period. These strategies may be effective in promoting fathers’ attitudes and knowledge about breastfeeding and increasing their self-efficacy to support their partners’ exclusive breastfeeding.

## Figures and Tables

**Table 1:** Descriptive Characteristics of the Participants (N = 215).

Characteristic	*n* (%)
Age, years	
20–29	83 (38.60)
30–39	108 (50.23)
40–49	23 (10.70)
≥50	1 (0.47)
Educational level completed	
<Bachelor’s degree	143 (66.51)
Bachelor’s degree and higher	72 (33.49)
Type of family	
Nuclear	94 (43.72)
Extended	121 (56.28)
Employment	
Unemployed	3 (1.40)
Employed	212 (98.60)
Income (Baht/Month)	
<10,000	24 (11.16)
10,000–19,999	115 (53.49)
20,000–29,999	49 (22.79)
30,000–39,999	19 (8.84)
≥40,000	8 (3.72)
Number of children	
1	165 (76.74)
>1	50 (23.26)

**Table 2:** Sociodemographic Factors Associated with Fathers’ Self-Efficacy to Promote Maternal Exclusive Breastfeeding (N = 215).

Variables	β	*SE*	*t*	*p*	95% Confidence Interval for β
Constant	55.28	7.22	7.66	0.00	41.05–69.51
Age, years	0.08	0.11	0.72	0.47	−0.14–0.29
<Bachelor’s degree	−0.38	1.37	−0.28	0.78	−3.08–2.32
Extended family	−3.26	1.26	−2.60	0.01 *	−5.74–−0.78
Employment	2.91	5.32	0.55	0.59	−7.58–13.39
Income Baht/Month	−0.01	0.01	−0.62	0.54	−0.01–0.01
Number of children = 1	−2.36	1.55	−1.52	0.13	−5.42–0.70

Note. A reference group, including unemployment, nuclear family, and bachelor’s degree and higher education, number of children > 1. * *p* < 0.05.

**Table 3:** Hierarchical Multiple Regression Analysis for Variables Affecting Fathers’ Self-Efficacy to Promote Maternal Exclusive Breastfeeding (N = 215).

Variables	β	*SE*	*t*	*p*	95% Confidence Interval for β
Model 1					
Constant	58.08	2.02	28.64	0.00	54.08–62.08
Extended Family	−3.38	1.23	−2.74	0.01 *	−5.82–−0.95
R^2^ = 0.034, Adjusted R^2^ = 0.03, F = 7.49					
Model 2					
Constant	28.39	6.37	4.46	0.00	15.84–40.95
Extended Family	−3.24	1.17	−2.75	0.01 *	−5.55–−0.91
Fathers’ attitude towards exclusive breastfeeding	0.24	0.05	4.89	0.00 *	0.15–0.34
R^2^ = 0.132, Adjusted R^2^ = 0.12, R^2^ change = 0.09, F = 16.09					
Model 3					
Constant	28.45	6.32	4.50	0.00	15.98–40.91
Extended Family	−3.35	1.17	−2.87	0.00 *	−5.66–−1.05
Fathers’ attitude towards exclusive breastfeeding	0.21	0.05	4.14	0.00 *	0.11–0.32
Fathers’ knowledge about exclusive breastfeeding	0.22	0.11	2.05	0.04 *	0.00–0.42
R^2^ = 0.149, Adjusted R^2^ = 0.14, R^2^ change = 0.02, F = 12.30					

* *p* < 0.05.

## Data Availability

The data presented in this study are available on request from the corresponding author. The data are not publicly available due to privacy.

## Public Involvement Statement

No public involvement in any aspect of this research.

## Guidelines and Standards Statement

This manuscript was drafted against the Strengthening the Reporting of Observational Studies in Epidemiology (STROBE; von Elm et al., 2007) reporting guidelines for observational studies.
